# COVID-19: The Impact on Cardiovascular System

**DOI:** 10.3390/biomedicines9111691

**Published:** 2021-11-15

**Authors:** Jozica Šikić, Zrinka Planinić, Vid Matišić, Tea Friščić, Vilim Molnar, Dorijan Jagačić, Lovro Vujičić, Neven Tudorić, Lana Postružin Gršić, Đivo Ljubičić, Dragan Primorac

**Affiliations:** 1 Department of Cardiology, Clinical Hospital Sveti Duh, 10000 Zagreb, Croatia; zrinkaplaninic@gmail.com (Z.P.); friscictea1@gmail.com (T.F.); 2 St. Catherine Specialty Hospital, 10000 Zagreb, Croatia; vid.matisic@svkatarina.hr (V.M.); vilim.molnar@svkatarina.hr (V.M.); djagacic@inet.hr (D.J.); lovro.vujicic@gmail.com (L.V.); Neven.tudoric@gmail.com (N.T.); divo.ljubicic@gmail.com (Đ.L.); draganprimorac2@gmail.com (D.P.); 3 University Hospital Centre Sisters of Mercy, 10000 Zagreb, Croatia; lana1505@yahoo.com; 4 Clinical Hospital Dubrava, 10000 Zagreb, Croatia; 5 Eberly College of Science, The Pennsylvania State University, University Park, State College, PA 16802, USA; 6 The Henry C. Lee College of Criminal Justice and Forensic Sciences, University of New Haven, West Haven, CT 06516, USA; 7 Medical School, University of Split, 21000 Split, Croatia; 8 Faculty of Dental Medicine and Health, Josip Juraj Strossmayer University of Osijek, 31000 Osijek, Croatia; 9 Faculty of Medicine, Josip Juraj Strossmayer University of Osijek, 31000 Osijek, Croatia; 10 Medical School, University of Rijeka, 51000 Rijeka, Croatia; 11 Medical School REGIOMED, 96 450 Coburg, Germany; 12 Medical School, University of Mostar, 88000 Mostar, Bosnia and Herzegovina

**Keywords:** SARS-CoV-2, cardiology, prevention, diagnostics

## Abstract

SARS-CoV-2 has been circulating in population worldwide for the past year and a half, and thus a vast amount of scientific literature has been produced in order to study the biology of the virus and the pathophysiology of COVID-19, as well as to determine the best way to prevent infection, treat the patients and eliminate the virus. SARS-CoV-2 binding to the ACE2 receptor is the key initiator of COVID-19. The ability of SARS-CoV-2 to infect various types of cells requires special attention to be given to the cardiovascular system, as it is commonly affected. Thorough diagnostics and patient monitoring are beneficial in reducing the risk of cardiovascular morbidity and to ensure the most favorable outcomes for the infected patients, even after they are cured of the acute disease. The multidisciplinary nature of the fight against the COVID-19 pandemic requires careful consideration from the attending clinicians, in order to provide fast and reliable treatment to their patients in accordance with evidence-based medicine principles. In this narrative review, we reviewed the available literature on cardiovascular implications of COVID-19; both the acute and the chronic.

## 1. Introduction

With over 240 million confirmed cases worldwide and over 4.5 million confirmed deaths since 12 October 2021, the COVID-19 pandemic is, without doubt, the most important event of the 21st century so far [1]. The coronavirus family is one of the major pathogens responsible for acute respiratory tract infections. Six known coronaviruses cause disease in humans, and the SARS-CoV and MERS-CoV viruses cause severe acute respiratory syndrome (SARS). In contrast, four other types that cause human diseases (HCoV-OC43, HCoV-229E, HCoV-NL63, and HCoV-HKU1) lead to mild upper respiratory tract infections, against which most adults have antibodies [2]. Earlier studies have found that serum antibodies begin to rise one week after contracting a coronavirus infection, reaching a peak after two weeks, and persisting for a long time and protecting individuals from reinfection from more severe forms of the disease [3]. The majority of known coronaviruses circulate among animals. It is still unclear why some types of coronaviruses switch to infecting humans, and many theories have been suggested; one of them warns that in some places, such as in very specific markets in Asia, contact occurs between animal species that do not regularly interact in nature. Such conditions create the potential for interactions between individual bacteria or viruses from different animal species, as well as for possible genetic recombination between them. This could lead to individual viruses assuming different behavioral patterns, regardless of the species involved. Since SARS-CoV-2 is a new virus that the human population has not been in contact with before, a population-wise lack of effective antibodies is our reality. Based on a mathematical approach from the reports of new cases from Wuhan and from traveling data from January 2019, it was calculated that the doubling time of spread was 2.4 days and that initially reported Rₒ from 2.2 to 2.7 was higher and is 4.7 to 6.6 [4]. The latest Rₒ value from the UK government reports indicates a relatively lower value for England, ranging from 1.2 to 1.5, with the latest infection growth rate from +3% to +7% per day [5]. A notable difference between SARS-CoV-2 and SARS viruses is that SARS-CoV-2 is able to replicate in the upper respiratory tract and that it reaches maximal viral load in nasal swabs much sooner, up to 5 days from the appearance of first symptoms with peak RNA concentrations up to 1000 times higher than in SARS [6]. Symptoms that arise due to COVID-19 infection mostly are caused by the infection of the respiratory epithelium and by the infection of angiotensin-converting enzyme 2 (ACE2) expressing tissues. Typical symptoms include cough, fever, shortness of breath, lost sense of smell and taste, nausea and diarrhea [7]. Apart from the usual symptoms found in most of COVID-19 infected individuals, certain conditions can be seen in patients that are critically ill or were admitted to the ICU. These include respiratory failure, coagulation abnormalities with subsequent thrombosis, thromboembolism, acute kidney injury and neurological symptoms with marked hypercoagulation and systemic inflammation seen in routine laboratory tests [7].

Asymptomatic individuals present a danger for the rapid spreading of the disease. Therefore, preventive measures to stop disease spreading should be equally applied to both symptomatic and asymptomatic individuals [8]. Knowing that a great proportion of infected individuals will develop COVID-19 it is of utmost importance for our patients that we have a firm understanding of its pathophysiology and its implications for various organ systems. In this review, we give an overview of cardiovascular implications of COVID-19 infection, the available diagnostics and management options of cardiovascular patients with COVID-19.

## 2. Pathophysiology of COVID-19

### 2.1. The Role of Angiotensin-Converting Enzyme 2 (ACE2) in the Pathophysiology of COVID-19

ACE2 is an important part of the angiotensin-renin-aldosterone system (RAAS) that controls blood pressure and fluid and electrolyte balance by inducing vasoconstriction or vasodilation, and which equilibrium is important for maintaining normal cardiac function [9,10]. Angiotensin-converting enzyme (ACE) converts angiotensin I (Ang-I) to angiotensin II (Ang-II) that activates angiotensin II type 1 receptor (AT1R) to promote vasoconstriction, fibrosis, and inflammation. ACE2 counterbalances the whole mechanism by targeting Ang-II and converting it to Ang 1–7, a vasodilator peptide that acts on Mas receptors to achieve vasodilatation, anti-inflammatory, antioxidative and antithrombotic effect, as well as reverse myocardial remodeling [10,11]. As ACE2 is the main entry receptor for SARS-CoV-2 infection in human tissues, it is important to know the biological features of the virus to better understand the underlying mechanisms of COVID-19, especially the cardiovascular implications of the disease [12].

SARS-CoV-2 is a single-stranded RNA virus consisting of four structural proteins known as spike (S), envelope (E), membrane (M) and nucleocapsid (N) proteins and belongs to *Betacoronavirus* genus together with SARS-CoV and MERS-CoV, which are also highly pathogenic coronaviruses in humans. Among structural proteins, spike (S) protein plays a crucial role in mediating the virus fusion with the host cell membrane and disease pathogenesis [12]. During SARS-CoV-2 infection, viral protein S binds to transmembrane ACE2 receptor, which triggers virus entry into the host cell [13]. Widespread expression of ACE2 as the SARS-CoV-2 entry receptor in numerous tissues, including the pulmonary, cardiac, renal and gastrointestinal systems, can explain multiorgan affection in COVID-19 [9,12]. The S protein has two subunits; the S1 subunit with receptor-binding domain (RBD) binds to angiotensin-converting enzyme 2 (ACE2), while the S2 subunit is responsible for the fusion between the membranes of the virus and the host cell. This process requires priming of viral S protein by cellular transmembrane protease serine 2 (TMPRSS2). It is important to note that infection with SARS-CoV-2 infection requires co-expression of ACE2 and TMPRSS2 in the same cell, again explaining viral tissue tropism. Besides the direct fusion of the cell membranes through priming of the S protein at novel furin cleavage sites specific for SARS-CoV-2, endocytosis is another possible entry pathway for the viral genome [12,13]. After viral and membrane fusion, the viral ssRNA is released in the cytosol, replicated and translated into viral structural proteins. So far, unknown viral mediators induce downregulation of ACE2 and upregulation of ADAM metallopeptidase domain 17 (ADAM-17) that sheds ACE2, a pathway further supported by increased cytokine levels [9]. The ACE2 downregulation induced by viral entry leads to prevailed angiotensin II effects that include inflammation, vasoconstriction, and increased risk of thrombosis. This can be partially explained by the host’s defense mechanism trying to limit viral proliferation, but conversely, diminished ACE2 activity in the heart enhances the susceptibility of heart failure [14].

Each step of the viral life cycle, including S protein priming by TMPRSS2 (serine protease inhibitor camostat mesylate), membrane fusion and endocytosis (antimalarial drug chloroquine and anti-influenza drug umifenovir) and RNA replication (antiviral agents favipiravir, remdesivir and ribavirin) is a potential therapeutic target [12]. 

Renin-angiotensin system inhibition is proven protective of the cardiovascular system because of its beneficial effect on blood pressure and ventricular remodeling. Since RAAS inhibitors such as angiotensin-converting enzyme inhibitors (ACEI) and angiotensin receptor blockers (ARBs) may increase the expression of ACE2 in type 2 alveolar epithelial cells in the lungs due to upregulation, the concern was raised that these medications could increase the risk of developing a more severe form of COVID-19, enhancing the susceptibility to viral entry through ACE2 binding of SARS-CoV-2. Conversely, ACEI and ARB decrease the production of angiotensin II, whose expression is responsible for lung injury by activating type 1 angiotensin receptor (AT1R), leading to the enhanced generation of angiotensin 1–7 which attenuates inflammation and fibrosis in the lungs [15]. A cohort study including 8.3 million people showed that neither ACE inhibitors nor ARBs are associated with increased risks of severe forms of COVID-19 and ICU admission [16]. Furthermore, a Wuhan study showed that neither ACEIs nor ARBs are associated with the severity or mortality of patients with hypertension hospitalized for COVID-19 [17]. There are still no findings that showed the protective effect of ACEIs or ARBs withdrawal in COVID-19 patients [12]. Since there is no clinical or scientific evidence supporting potential harmful effect of ACEIs or ARBs so far, both the European Society of Cardiology (ESC) *Guidance for the Diagnosis and Management of CV Disease during the COVID-19 Pandemic* and The Council on Hypertension of the European Society of Cardiology recommend continuation of usual antihypertensive therapy if indicated [18].

### 2.2. Endotheliopathy and Microvascular Injury

In the last few decades, many studies were published linking endothelial dysfunction with cardiovascular diseases [19]. The endothelium regulates the intensity of local inflammation by controlling leukocyte adhesion and their entry into various tissues [20]. Moreover, endothelium serves as an autocrine and a paracrine cell that secretes various cytokines and locally acting substances [21]. Known cardiovascular risk factors, including smoking, hypercholesterolemia, and aging are associated with endothelial dysfunction; these and other risk factors lead to increased production of vasoconstricting substances and prothrombotic factors and decreased production of antithrombotic factors. Endothelial dysfunction is characterized by increased leukocyte-endothelial interaction, the release of inflammatory mediators and a procoagulant state [19]. Besides being caused by cardiovascular risk factors, endothelial dysfunction that promotes local inflammation can be also caused directly by inflammation itself. Disorders such as autoimmune diseases and systemic inflammatory response triggered by the release of systemically and locally acting inflammatory cytokines promote further deterioration of endothelial function [22]. These risks then lead to the formation of atherosclerotic plaques, vessel damage, or thrombus formation. Reversal of endothelial dysfunction is a key point in today’s treatment of diseases such as diabetes, hypertension, hyperlipidemia and many others [19]. It seems that pericytes (mural endothelial cells) are having an important role during the inflammatory process, most likely via different cytokines, chemokines, etc. [23]. Furthermore, it has been demonstrated that pericyte-specific vascular expression of SARS-CoV-2 receptor ACE2 is playing a critical role in microvascular inflammation and hypercoagulopathy in COVID-19 patients [24].

Epidemiologic analysis of over 20,000 COVID-19 patients admitted to UK hospitals found that comorbidities including chronic cardiac disease, non-asthmatic chronic pulmonary disease, chronic kidney disease, liver disease and obesity were associated with higher mortality in hospital [25]. Interestingly, most of these states correlate with chronic endothelial dysfunction, therefore we can hypothesize that the acute damaging effect of SARS-CoV-2 on the endothelium aggravates this chronic condition, ultimately leading to a severe disease manifestation and worse outcomes. Cytokines, destabilized coronary plaque, severe hypoxia, and viral infection of pericytes could contribute to myocardial damage, formation of microscopical thrombi and emboli, and in the lymphatic vessels, lymphocyte leakage causing lymphopenia, which is frequently observed in COVID-19 [26]. The endothelium can be a target for infection by microorganisms, such as Rickettsia or Bartonella species and if not directly infected by a microorganism, an acute inflammatory response caused by a microorganism can generate endothelial dysfunction systemically [27]. The ACE2 receptor, which is vastly concentrated in lung alveolar cells, can also be found on the endothelium of blood vessels and cardiac myocytes [28,29]. Therefore, it must be hypothesized that novel coronavirus can infect the endothelial cells and therefore cause endothelial dysfunction by a local inflammation (endothelitis). If not directly infected by viral particles, the systemic inflammatory response generated by severe infection could cause a generalized endothelial dysfunction.

The endothelium can also cause and contribute to a rapidly spreading cytokine storm that is formed when the body’s protective and antioxidative system cannot suppress the spread of cytokines and prooxidative substances across the body as a whole. Higher levels of interleukin 6 (apart from higher levels of all other cytokines that usually accompany cytokine storm) are associated with shorter survival [30]. Whereas it was speculated during the start of the COVID-19 pandemic that anti-IL6 drugs could perhaps alleviate or even stop the symptoms of critically ill patients, a recent study by Rosas et al. showed that there was no significant difference regarding clinical status or mortality between the tocilizumab-given and placebo-given group [31]. In contrast, a study performed by Gupta et al. showed that a group of critically ill patients who were given tocilizumab in the first 2 days of ICU admission had a lower in-hospital risk of mortality, but it must be underlined that tocilizumab treated patients were generally younger and had fewer comorbidities [32]. A study conducted on autopsied testicular and epididymal specimens of deceased COVID-19 patients showed histologically increased levels of interleukin-6, TNF-𝛼 and MCP-1, decreased sperm concentration and a wide presence of microinflammation-caused interstitial edema, congestion, red blood cell exudation compared to control males [33]. Besides higher levels of cytokines and prooxidative substances, markers of endothelial and platelet activation are likewise increased in those suffering from COVID-19 infection, especially in patients with severe disease courses. Von Willebrand factor and P-selectin levels were markedly increased in a study conducted by Goshua et al. in both ICU patients and non-ICU patients. In addition, levels of VWF and soluble thrombomodulin correlated with patient mortality [34].

The increasing body of evidence suggests that chronic endothelial dysfunction is the crucial pathophysiological mechanism leading to a severe course of COVID-19 and worse patient outcomes. These findings should guide the patient approach in the future. Multiple studies conducted during the COVID-19 pandemic tried to find a correlation between certain angiogenic cytokines and potentiation of the systemically and locally produced inflammation. A study by a group of authors from France found that increased levels (over 5000 pg/mL) of angiopoietin 2, a marker of endothelial dysfunction, is a good predictor of disease outcome and ICU admission with the sensitivity of 80.1% and specificity of 70% [35]. Another study conducted by Alay et al. showed that serum levels of markers such as angiopoietin 2 and surfactant protein D are associated with clinical severity of COVID-19 patients. Patients with severe/critical COVID-19 disease had highest measured levels of given markers whereas those that were asymptomatic or had mild disease had lesser count. Cut-off values were determined statistically for each of the markers for differentiating COVID-19 cases and healthy individuals, for SP-D cut-off value was 37.7 ng/mL and for angiopoeitin 2 was 4208.3 pg/mL. Surfactant protein D is a protein released via alveolar type II cells that regulates pulmonary inflammatory response and defense [36]. A study conducted by Villa et al. showed that increased levels of angiopoetin 2 measured on the 3rd day of hospitalization are a good predictor with in-hospital mortality whereas increased levels on the 10th day were a predictor of nonresolving chronic pulmonary condition [37]. A retrospective study by Kweon et al. has showed that a myriad of laboratory markers such as procalcitonin, D-dimers, angiopoietin-2, tumor necrosis factor-α, endocan 4-day and 7-day increasment were significantly higher in the weaning failure group. Weaning failure was defined as death, discharge with oxygen therapy before day 28 and requirement of oxygen support as of day 28. ARDS and mechanical ventilation were more prevalent in the weaning failure group, and the quick Sequntial Organ Failure Assessment (qSOFA) score was higher in the same group. The best predictor for weaning failure was the 7-day increase in endocan with 78% sensitivity and 79% specificity [38]. Encouraging results such as these showcase the potential for a more precise treatment algorithm in the future, which would enable better treatment individualized for patients. Considering the future, it is plausible that pulmonary fibrosis could affect the patients’ daily life, but to which extent this is a case for mild and moderate cases is yet to be determined. Therefore, careful monitoring of asymptomatic cases and younger patient groups that are more physically active and use more of their cardiac and pulmonary functional capacity, such as athletes, should be considered given our past knowledge, high-intensity sport and physical activity suppress the immune system and stimulate the production of oxidants [39].

### 2.3. Coagulopathy and Thromboembolic Issues in COVID-19

**Figure 1:**
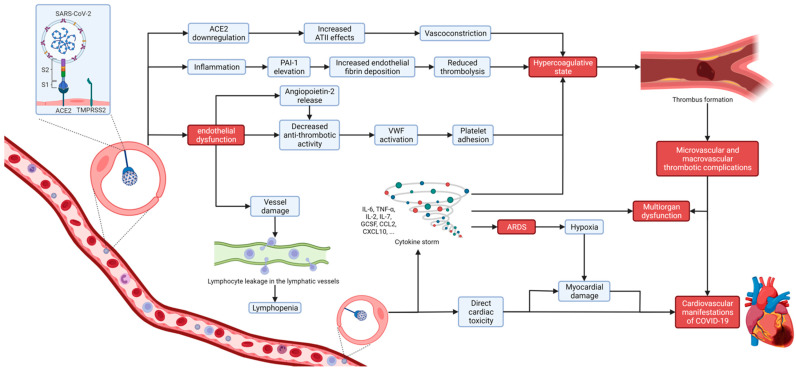
Schematic representation of COVID-19 induced endothelitis. SARS-CoV-2 binding to ACE2 on endothelial cells induces inflammatory-mediated changes and endothelial dysfunction that causes the characteristic hypercoagulative state. In turn, causing micro- and macrovascular thrombotic events often seen in severe COVID-19 patients. Created in Biorender.com (accessed on 6 November 2021). TMPRSS2—transmembrane protease serine 2; ACE2—angiotensin-converting enzyme 2; ATII—angiotensin II; PAI1—plasminogen activator inhibitor 1; VWF—von Willebrand factor; IL-6—interleukin 6; IL-2—interleukin 2; IL-7—interleukin 7; TNF-α—tumor necrosis factor-α; GCSF—granulocyte colony-stimulating factor; CCL2—chemokine (C-C motif) ligand 2; CXCL10—C-X-C motif chemokine 10; ARDS—acute respiratory distress syndrome.

Coagulopathy and thromboembolic complications are common characteristics of COVID-19, especially in critically ill patients, but can cause death even in asymptomatic patients. COVID-19 associated coagulopathy (CAC) is based on a complex thromboinflammatory process including endotheliopathy due to direct endothelial infection with SARS-CoV-2 and the indirect damage caused by inflammation resulting in both microvascular and macrovascular thrombotic complications that may lead to multiorgan dysfunction (Figure 1) [40]. The prothrombotic state in COVID-19 may be explained by several mechanisms that include direct damage (microvasculitis), indirect damage (downregulation of ACE2 receptor, hypoxia, and DIC) and possibly even behavioral mechanisms (prolonged bed restriction, especially for ICU patients, and social isolation) [41]. SARS-CoV-2 infects endothelial cells through ACE2 receptors causing direct damage, cell apoptosis and decreased antithrombotic activity of the disrupted luminal surface leading to subendothelial tissue exposure which then activates von Willebrand Factor (VWF), platelet adhesion, and the coagulation cascade. In addition to this, inflammation causes plasminogen activator inhibitor 1 (PAI-1) elevation, leading to increased endothelial fibrin deposition and reduced thrombolysis [42,43]. This disruption of the normal homeostasis of vascular endothelial cells results in microangiopathy, microvascular clot formation, and systemic hypercoagulability that may cause major thromboembolic complications, including pulmonary embolism [40]. ACE2 downregulation due to viral entry leads to increased effects of angiotensin II and decreased conversion to angiotensin 1-7, resulting in suppressed nitric oxide production, vasoconstriction, and platelet adhesion, further contributing to prothrombotic state [44]. Excessive proinflammatory cytokine production causing cytokine storm in severe forms of COVID-19 can induce hemophagocytic lymphohistiocytosis (HLH) and macrophage activation syndrome (MAS), which also promotes coagulation disorder [45].

SARS-CoV-2 induced hypercoagulability, platelet activation, and endothelial dysfunction have similarities with disseminated intravascular coagulation (DIC) as seen in bacterial-induced sepsis, but with some differences [46]. Typical CAC can be diagnosed by increased D-dimer, which is the most common finding, then elevated fibrinogen and von Willebrand factor (VWF) levels, while prothrombin time (PT), activated partial thromboplastin time (aPTT), and platelet count are usually within the normal range [40]. Abnormally increased D-dimer levels according to studies were found in 46.4%, with a prevalence of 43% in non-severe patients compared with 60% in critically ill ICU patients [47]. Except for D-dimer, VWF and factor VIII are typically increased in CAC, as they are released from the Weibel–Palade body of endothelial cells as a response to SARS-CoV-2 infection, but their monitoring is limited in everyday clinical practice [48,49,50]. Goshua et al. reported significantly higher levels of VWF antigen/antibody, FVIII activity, and thrombin-antithrombin complex (TAT) in severe cases of COVID-19 and, these are likely associated with a poor prognosis [34]. Moreover, angiopoietin-2 released from damaged endothelial cells is also associated with hypercoagulability and is a relevant predictive factor for ICU admission in COVID-19 patients [35,51]. Thrombocytopenia is not a common initial finding in COVID-19 [52]. According to one study, platelet counts of less than 100 × 10^9^/L were reported in only 8% of ICU and 4% in non-ICU patients at admission, while another study that compared the platelet count between COVID-19-associated ARDS patients and non-COVID-19 ARDS patients showed no significant difference in platelet count [53,54]. The lack of thrombocytopenia differs CAC from disseminated intravascular coagulopathy (DIC) that is typically consumptive coagulopathy [40]. D-dimers and fibrinogen levels should be regularly monitored, and all hospitalized patients should be treated with thromboembolism prophylaxis, usually low molecular heparin (LWMH) if no contraindications [40].

### 2.4. SARS-CoV-2-Induced Immune Dysregulation of the Cardiovascular System and Cytokine Storm

SARS-CoV-2 infection can cause a maladaptive immune response due to excessive proinflammatory cytokine production leading to cytokine release syndrome (CRS) or cytokine storm in severe COVID-19 contributing to mortality, as described in SARS and MERS. Since both monocytes and macrophages express ACE2, SARS-CoV-2 infection results in the activation and transcription of proinflammatory genes [55]. Cytokine storm, as observed in severe forms of COVID-19, is a consequence of immune system overactivation caused by infection leading to hyperproduction of numerous cytokines and chemokines, such as IL-6 IL-2, IL-7, granulocyte colony-stimulating factor, C-X-C motif chemokine 10 (CXCL10), chemokine (C-C motif) ligand 2, and tumor necrosis factor-α (TNF- α) whose uncontrolled release into circulation contribute to the development of acute respiratory distress syndrome, multiorgan dysfunction and fatal outcome [53,56,57,58]. Exaggerated circulation of proinflammatory cytokines causes endothelial damage, leading to vasoconstriction, coagulation disorder and thrombus formation [59,60,61]. Interleukin-6 (IL-6), produced mainly by activated macrophages, seems to be the key cytokine involved in SARS-CoV-2 mediated cytokine storm and its levels correlate with mortality in critically ill patients, making IL-6 an important biomarker of disease severity and prognosis [58,62]. Many proinflammatory cytokines were found to be significantly increased in critically ill COVID-19 patients, but elevated levels of IL-6 were the most frequently detected [55]. IL-6 plays an important role in the pathogenesis of coronary artery disease. Its chronically increased levels induced by obesity and smoking strongly correlate with hypertension, dyslipidemia and glucose alterations promoting atherosclerosis, explaining the positive association between elevated levels of IL-6 and risk of cardiovascular mortality [62,63]. Since its important role in SARS-CoV-2 induced cytokine release syndrome, the IL-6 signaling pathway has become a promising therapeutic target for severe forms of COVID-19. There are ongoing studies with IL-6 receptor blockers, such as tocilizumab, sarilumab, olokizumab and sirukumab, that are still unproven in terms of their benefit in treating COVID-19 patients, although tocilizumab has proven to reduce the proatherogenic effects of IL-6, therefore suggesting it may improve survival among COVID-19 patients [62,64]. In COVID-19, besides high cytokine levels, lymphocyte count is usually reduced with elevations in levels of D-dimer, C-reactive protein (CRP), ferritin, and procalcitonin that also support the development or continuation of cytokine storm in COVID-19 patients [47,65]. Furthermore, there is a marked decrease in circulating T and B lymphocytes, particularly in severe forms of COVID-19 infection due to aberrant activation of monocytes and macrophages, and an increase of neutrophils [55]. According to numerous clinical reports, lymphopenia, especially the decreased amount of CD4+ and CD8+ T cells, is closely related to disease severity of COVID-19, and CD8+ T cells appear to be an independent predictor for COVID-19 severity, as well as their dysfunction may contribute to cardiovascular disease development [18,66,67]. When it comes to T cells, CD4+ T cells are helper cells that help B cells in the production of antibodies, while CD8+ T cells are killer cells that mediate the elimination of infected cells. Lymphopenia is a common finding in COVID-19, affecting both CD4+ T cells and CD8+ T cells, more significantly in critically ill patients, considering that T cell response may be either diminished or overactivated. Since the majority of COVID-19 convalescents develop a strong T cell response, this could be a foundation for long-term immunity, as some of them have a memory phenotype [55,68].

### 2.5. Direct SARS-CoV-2 Cardiomyocyte Toxicity

Cardiovascular disease (CVD) has an important role in COVID-19, not only as a risk factor but also as a predictor of mortality. Preexisting CVD was noted in previous studies with a variable prevalence from 2% to 40%, although new reports suggest that the prevalence of CVD in COVID-19 tends to be higher than in the general population, especially in severe and critical cases [69,70]. A recent meta-analysis including 6270 COVID-19 patients found that 41.1% had associated comorbidities such as hypertension (20.9%), diabetes (9.96%), and CVD (4.8%). The history of CVD was associated with an increased risk of severe COVID-19 with OR 4.81 (95% CI: 3.43–6.74), where the severity was defined as the need for hospitalization, admission to the ICU, and death. Additionally, a significant association with the disease severity was found in patients with cerebrovascular disease, chronic lung disease, cancer, diabetes, and hypertension [71]. High mortality risk in COVID-19 patients with a history of CVD can be due to the possibility that those patients are more prone to hemodynamic deterioration in severe and critical cases because of preexisting ventricular dysfunction. System inflammatory response might turn chronic coronary artery disease (CCS) into acute coronary syndrome (ACS) due to hypercoagulative state, atherosclerotic plaque rupture and subsequent thrombotic events [72]. A large study from China with 44,672 confirmed cases reported an overall case fatality rate of 2.3% and a much higher fatality rate of 10.5% for patients with CVD [73]. Male sex and advanced age are also associated with higher mortality [74].

The underlying mechanisms of cardiac injury are still not clearly understood, but it has been hypothesized that they are most likely multifactorial. Direct cardiotoxicity of SARS-CoV-2 can occur due to the ACE2 expression on myocardial cells and cardiac pericytes inducing capillary endothelial and microvascular dysfunction. Downregulation of ACE2 has a potential protective role for the heart by negatively regulating the renin-angiotensin system. Hypoxemia, as a consequence of lung involvement, can contribute to direct cardiac injury [75,76].

The SARS-CoV-2 infection causes a maladaptive immune system response and cytokine storm leading to excessive proinflammatory cytokines release that depresses myocardial function immediately through activation of the neural sphingomyelinase pathway and subacutely via nitric oxide-mediated blunting of beta-adrenergic signaling. An excess of proinflammatory cytokines could result in vascular inflammation, plaque instability, myocardial inflammation, hypercoagulable state such as DIC, and supply–demand mismatch [58,75]. 

Cardiovascular manifestations of COVID-19 include myocardial injury, myocarditis, ACS, arrhythmias, heart failure, arterial and venous thromboembolism (VTE), and cardiogenic shock [69].

## 3. Cardiovascular Manifestation and Clinical Course of COVID-19

### 3.1. Arrhythmias

There is growing evidence showing that arrhythmias are a major cardiovascular manifestation in COVID-19. Mechanisms for arrhythmogenicity in viral infections include altered intercellular signaling, ion channel dysfunction, electrophysiological and structural remodeling due to interstitial edema and cardiac fibrosis resulting in repolarization abnormalities and action potential conduction abnormalities [77]. Arrhythmia in COVID-19 can be secondary to electrolyte imbalance (especially hypokalaemia and hypomagnesemia), pulmonary disease, medication side effects, activated protein kinase C (PKC), or direct oxidized Ca^2+^/calmodulin-dependent protein kinase II (CAMKII) [43]. A recently published study compared COVID-19 ICU patients with ICU patients who were COVID-19 negative and showed that atrial tachyarrhythmia is associated with increased mortality in critically ill patients with COVID-19 (OR 5.0, 95% CI 1.9–13.5) [78]. Interestingly, COVID-19 patients who were mechanically ventilated were more vulnerable to hemodynamic compromise after atrial tachyarrhythmia onset than COVID-19 negative patients. The incidence of arrhythmias has been associated with disease severity. According to a study from Wuhan, China, patients with severe/critical COVID-19 had more atrial arrhythmias compared with those with non-severe symptoms. When they compared COVID-19 ICU patients with patients who were admitted to the ICU due to bacterial pneumonia in the pre-COVID era, those with COVID-19 had higher total, mean, and minimum heart rates, and no significant difference in the incidence of arrhythmias, including premature atrial/ventricular contractions, atrial fibrillation, nonsustained ventricular tachycardia, and atrioventricular block [79]. Studies from China, the USA and Europe showed that non-specifical arrhythmia was the most common in COVID-19 patients. To be more precise, the overall incidence was about 17%, non-specifical arrhythmia were 12%, atrial fibrillation 8%, conduction abnormalities 11%, premature contractions 9% and about 3% ventricular tachycardia/fibrillation [80].

A global survey of a total of 1197 electrophysiology (EP) professionals from 76 countries and 6 continents reported atrial fibrillation as the most common tachyarrhythmia in COVID-19 patients (in 21% of the reports), and severe sinus bradycardia and complete heart block as the most common bradyarrhythmias (in 8% and 5.9% of the reports, respectively). Regarding life-threatening arrhythmias, ventricular tachycardia/ventricular fibrillation arrest and pulseless electrical activity were reported by 4.8% and 5.6% of respondents, respectively [81]. The use of chloroquine, hydroxychloroquine (HCQ) and azithromycin in COVID-19, because of their potential ability to interfere in the cell endocytosis of the virus, their immunomodulatory and anti-inflammatory effects, raised the question of their safety regarding their proarrhythmic effects and direct myocardial toxicity via impairment in intracellular degradation and accumulation of pathological metabolic products such as phospholipid and glycogen [82]. Chloroquine and HCQ can cause irreversible cardiomyopathy and arrhythmias, such as high-grade atrioventricular block, bundle branch block, QT prolongation and torsades de pointes (TdP). Furthermore, American Heart Association has listed chloroquine and HCQ as agents which can cause direct myocardial toxicity manifesting as restrictive or dilated cardiomyopathy and exacerbates underlying myocardial dysfunction [82]. As mentioned in a European Society of Cardiology review paper about cardiac safety of COVID-19 drug therapy, Favipiravir, Lopinavir/Ritonavir, Azithromycin and Piperacillin-Tazobactam can also cause prolonged QT and TdP. Patients with COVID-19 receiving those agents should be closely monitored, especially when combining more agents at a time or having congenital long QT, electrolyte imbalances, preexisting heart disease, and renal or hepatic dysfunction [83]. The last update of Survival Sepsis Campaign COVID-19 Guidelines stated a strong recommendation against the use of hydroxychloroquine for the treatment of severe or critical COVID-19. The rationale behind is that for now the use of HCQ in hospitalized adults with COVID-19 did not reduce mortality or the need for invasive ventilation, but instead increased adverse events, and even the risk of death [84].

### 3.2. Acute Cardiac Injury and Acute Coronary Syndrome

Myocardial injury, defined by the Fourth Universal Definition of Myocardial Infarction as cardiac troponin (cTn) concentrations >99th percentile upper reference limit (URL) is a common and consistent finding among patients with COVID-19 infection. An increase in cTn is caused by acute non-ischemic myocardial injury, ACS or a chronic myocardial injury [85,86].

According to a cohort study on 416 patients with COVID-19, acute myocardial injury was found in 19.7% of patients during hospitalization, and it was an independent risk factor for in-hospital mortality [87]. In a meta-analysis including 10 studies with a total of 3118 patients, the prevalence of myocardial injury during hospitalization was 15% to 44%. Furthermore, patients with elevated troponin levels had a significantly higher mortality risk than those with normal troponin levels, with an unadjusted odds ratio of 21.15 suggesting that elevated cTn may be considered as a mortality risk among COVID-19 patients [72].

Myocardial injury associated with COVID-19 can be seen as a reflection of the disease severity because it is related mainly to indirect mechanisms of cardiac tissue damage such as to non-ischemic myocardial processes due to hypoxia, sepsis, systemic inflammation, VTE, and cardiac adrenergic hyperstimulation during cytokine storm [72].

A study on 187 patients with COVID-19 showed a significant increase in mortality during hospitalization in those who had elevated cTn (37.5%), especially when combined with underlying CVD (69.44%). Patients with elevated cTn levels had more frequent malignant arrhythmias, and indications for mechanical ventilation [88]. In an early study from China that enrolled 101 cases, the acute myocardial injury was present in 15.8% of COVID-19 patients who were older, with a higher prevalence of preexisting cardiovascular disease, more frequently classified as severe/critical, and was more likely to require admission to ICU, mechanical ventilation and vasoactive agents [89]. Because of the higher prevalence of myocardial injury than that of underlying CVD, it can be speculated that SARS-CoV-2 affects the cardiovascular system, not only through exacerbation of the preexisting state but also directly causing myocardial inflammation due to extensive release of proinflammatory cytokines, which, in some cases, leads to fulminant myocarditis [90]. According to the current European Society of Cardiology Guidance for the Diagnosis and Management of CV Disease, during the COVID-19 pandemic admission and peak troponin appear to be predictors for outcomes in COVID-19 patients. The use of serial cTn could facilitate risk stratification and help make decisions about when to use imaging [18].

Possible mechanism of myocardial injury in COVID-19 involves ischemic etiology as in ACS. Plaque rupture and subsequent thrombus formation due to activated macrophages, platelets and coagulation cascade as a part of the systemic immune response lead to myocardial infarction (MI) in the affected vessel irrigation territory (type I), i.e., ST-elevation myocardial infarction (STEMI). Although it seems plausible, only a few cases of STEMI in patients with COVID-19 have been reported [91]. Supply–demand mismatch in patients with underlying coronary artery disease is believed to cause the majority of MI cases (type II) because of hypoxemia, acidosis, hypotension, and arrhythmias that accompany severe/critical COVID-19 presentation [90]. A recently published case series described three patients with STEMI and a coronary angiogram consistent with type II MI [92]. Moreover, a case of STEMI in the setting of COVID-19 was described as caused by microvascular thrombi in the absence of epicardial coronary artery disease [93]. An observational study that included 39 COVID-19 patients with STEMI showed higher levels of troponin T and lower lymphocyte count, but elevated D-dimer and C-reactive protein compared to patients without COVID-19. There were significantly higher rates of multivessel thrombosis, stent thrombosis, a requirement of higher use of glycoprotein IIb/IIIa inhibitors, higher doses of heparin to achieve therapeutic activated clotting times, suggesting a higher thrombus burden. Levels of D-dimer were found to correlate with thrombus grade, myocardial blush grade, and levels of heparin requirement during the primary PCI procedure. Also, patients with STEMI and COVID-19 had longer in-patient admission and higher rates of intensive care admission [94].

Although COVID-19 has a great potential to cause ACS or exacerbate previous chronic conditions, there are reports from Italy, Spain, and the USA showing a significant reduction in hospitalization for ACS (40–48%), and also a reduction in percutaneous coronary intervention (38–40% for STEMI, 57% for diagnostic procedures) [95,96,97]. The report from Italy also showed a 26.5% reduction in STEMI admissions and a 65.4% reduction in NSTEMI admissions with a disproportionately greater decrease in STEMI reductions for women (41.2%), than for men (25.4%). The STEMI case fatality rate increased to 13.7% from 4.1% in 2019 and the rate of major complications increased to 18.8% from 10.4% in 2019 [95]. In central Germany, comparing lockdown in 2020 to 2019 cardiac death, pulmonary embolism, and stroke rose about 8%; however, fatal pulmonary embolism was about 11% higher [98]. Some of the reasons for this trend could be avoidance of medical facilities because of social distancing, concerns of contracting COVID-19 in healthcare settings, and less physical activity. Conversely, the rate of out-of-hospital cardiac arrest increased. A study from Italy showed a 58% increase in out-of-hospital cardiac arrest compared with the pre-COVID-19 period. The cumulative incidence of out-of-hospital cardiac arrest in 2020 was strongly associated with the cumulative incidence of COVID-19. Interestingly, there was a decrease of 15.6% in cardiopulmonary resuscitation from bystanders possibly showing the side effects of social distancing [99]. The even greater difference was found in a population-based cross-sectional study from New York City with the number undergoing resuscitation 3-fold higher during the 2020 COVID-19 period compared with the non-COVID-19 period in 2019. Patients suffering an out-of-hospital cardiac arrest during 2020 were older, nonwhite race/ethnicity, and more likely to have specific comorbidities, such as diabetes and hypertension. There was a significant increase in non-shockable presenting rhythms and a substantial reduction in return of spontaneous circulation (ROSC) [100]. It is becoming evident that not only COVID-19 itself, but the social and economic circumstances that follow the pandemic have a significant impact on cardiovascular health, bringing new challenges for the healthcare system in the post-COVID-19 era.

### 3.3. Vasculitis/Coagulopathy

A review by Zhang et al. highlighted the possibility that SARS-CoV-2 infection can generate vasculitis and thrombosis in severe COVID-19 patients. Pathological examination in these patients showed congested and edematous blood vessels of alveolar septa with infiltration of monocytes and lymphocytes. Small vessels showed hyperplasia, wall thickening, lumen stenosis, occlusion and focal hemorrhage. Moreover, some patients tested positive with antiphospholipid antibodies and were associated with severe thrombosis [101].

A study from China analyzing postmortal renal histopathology showed no findings of viral particles inside the endothelium of glomeruli, whereas podocytes of glomeruli and proximal renal tubules cells did show viral particles as a sign of an infection. Even though no sign of endothelium infection was recorded, researchers found signs of obstruction in peritubular capillaries caused by erythrocyte stagnation. This can be caused by a locally induced inflammation around the infected podocytes and proximal renal tubules [102]. In the myocardium, the cells that express ACE2 the most are pericytes, who interact the most with endothelial cells in the human heart. This finding points to the fact that the infection of pericytes by SARS-CoV-2 causes endothelial dysfunction and could be the reason for the observed correlation of severe disease course and history of cardiac illness. Another key fact in this consideration is that ACE2 is expressed in greater quantities in patients suffering from heart failure, making them more susceptible to infection [28].

Once a microorganism enters host endothelial cells it starts a chain reaction leading to a procoagulant state with the risk of causing disseminated intravascular coagulation (DIC). This was observed in autopsy reports of patients who died from COVID-19, where the pathohistology revealed exudative/proliferative diffuse alveolar damage, with intense epithelial viral cytopathic effects involving alveolar and small airway epithelium, and little lymphocytic infiltration. Thrombosis of small vessels alongside endothelial tumefaction with an increased count of megakaryocytes was observed in 8 out of 10 patients. These findings are highly suggestive of a hypercoagulative state in critically ill patients [103].

A study from the Netherlands followed 184 ICU patients diagnosed with SARS-CoV-2 pneumonia, during their hospital stay and found a high incidence (31%) of thrombotic complications. These included pulmonary embolism, as the most frequent (81%), other venous thromboembolic events and arterial thrombotic events, all of which were ischemic strokes. The authors also emphasized that venous thromboembolism could have had a higher incidence since strict isolation made VTE screening more difficult [104]. In recent research published in JAMA, the authors reported that up to 79% of patients admitted to the ICU had signs of deep vein thrombosis when a venous ultrasonographic examination was performed [105].

A study by Oxley et al. recorded 5 cases of large vessel strokes in patients younger than 50 years of age in New York hospitals. The authors emphasized that the number of people younger than 50 years old, who suffered from large vessel stroke 12 months before the COVID-19 epidemic was much smaller in opposition to the recorded cases. Systemic endothelial dysfunction was thought of as causative for two COVID-19 patients who suffered an ischemic stroke in a study by Barrios-López et al. [106]. A study comparing patients admitted with COVID-19 infection versus those admitted with influenza virus infection showed that whereas only 3 out of 1486 patients (0.2%) with influenza had an ischemic stroke, 31 out of 1916 patients (1.6%) with COVID-19 had it, likewise. Out of 31 patients hospitalized for COVID-19, 8 patients were admitted because of stroke symptoms and the rest developed the symptoms while hospitalized [107].

A study from Bergamo province, Italy reported a 30-fold increased incidence of Kawasaki-like disease in children. The study included all the children diagnosed with a Kawasaki-like disease divided into two groups. The first group included 19 patients who were diagnosed with Kawasaki-like disease between 1 January 2015, and 17 February 2020, and the second group, which included 10 patients, between 18 February and 20 April 2020. The second group was more likely to develop cardiac involvement, Kawasaki disease shock syndrome, macrophage activation syndrome during the disease course, and the need for adjunctive steroid treatment was likewise higher. SARS-CoV-2 infection was proven in the second group via serology in 8 of 10 patients, with either using IgM or IgG titers, whereas nasopharyngeal swab was positive in 2 out of 10 patients. This finding can be hypothesized that Kawasaki-like disease is a late-onset event in disease course compared with primary infection [108]. Dermatological manifestations can also be caused by vascular pathology and underlying endothelial dysfunction. Two case reports from Italy recorded skin involvement in subjects with moderate-to-severe lung infection. The first patient developed a widespread urticarial rash, whereas the second patient first presented with vasculitic purpura of legs, which evolved into an erythematous rash [109]. A retrospective study from France reported that 7 of 14 patients included in the study had vascular lesions of the skin, whereas the other 7 patients had inflammatory lesions. Reported vascular lesions were livedo, violaceous macules, non-necrotic purpura, necrotic purpura, chilblain, chilblain with Raynaud’s phenomenon and an eruptive cherry angioma. Reported inflammatory lesions were exanthema (4 patients), chickenpox-like vesicles (2 patients) and cold urticaria (1 patient). Pathophysiology of the lesions could include immune dysregulation, vasculitis, vessel thrombosis, or neo-angiogenesis [110]. Vascular angiogenesis was proven postmortem in lungs analyzed during autopsy in a study by Ackermann et al. which proved that in lungs of COVID-19 critically ill patients angiogenesis (mostly intussusceptive) produced 2.7 times larger amounts of vessels contrary to lungs of deceased from equally severe influenza infection [111].

### 3.4. COVID-19-Related Myocarditis

Acute non-ischemic myocardial injury due to primary cardiac illness can occur in COVID-19 patients presenting with myocarditis, stress cardiomyopathy and acute heart failure. The epidemiology and pathogenesis of myocarditis in COVID-19 are currently unknown. The largest study to date regarding acute myocarditis in COVID-19 is a systematic review of 38 reported cases which does not reflect the true incidence of myocarditis in COVID-19. It is a challenge to differentiate between myocardial injury in severe/critical COVID-19 and acute myocarditis, especially because of the lacking availability of diagnostic procedures, such as endomyocardial biopsy (EMB) or cardiac magnetic resonance (CMR), during the COVID-19 pandemic [112]. The possible mechanism of myocardial damage is the pathway via ACE2 receptors and a hyperimmune response. Early studies from China reported five cases of myocardial damage resulting in circulatory failure as a possible consequence of fulminant myocarditis, although the diagnosis of myocarditis was not confirmed [58]. Suspicion of acute myocarditis should be raised when the levels of cTn do not correlate with the hyperinflammatory state. High levels of cTn and N-terminal Pro-B-type Natriuretic Peptide (NT-pro BNP) accompanied with a generalized increase in inflammatory markers are a sign of multiorgan failure rather than acute myocarditis [112]. There are no pathognomonic electrocardiographic changes in COVID-19 myocarditis. The most common ECG changes in COVID-19 are ST-segment abnormalities (40%), followed by cardiac arrhythmias (38%) including sinus tachycardia, sinus bradycardia, conduction blocks, atrial premature complex, atrial tachycardia, atrial fibrillation, and ventricular premature complex [113]. Echocardiographic features of COVID-19 myocarditis are global and regional hypokinesia with a decreased left ventricular ejection, and an increase in wall thickness suggesting edema. A normal echocardiographic exam is possible, especially in the early stages [112]. Cardiac magnetic resonance (CMR) imaging can show increased T2 values and positive short T1 inversion recovery (STIR), subepicardial or transmural late gadolinium enhancement (LGE) along with hypokinesis, left ventricular dysfunction and pericardial effusion [114]. In a cohort study including 39 consecutive autopsy cases from patients positive for SARS-CoV-2, the virus RNA was confirmed in 24 of 39 patients (61.5%). None of the patients in the study were diagnosed with fulminant myocarditis, and there were no massive cell infiltrates or necrosis [115]. A study including 104 EMB of patients with suspected myocarditis or unexplained heart failure detected SARS-CoV-2 genome in 5 cases, and only 2 of them met the Dallas criteria for myocarditis [116]. In an international multicentre study assessing cardiac tissue from the autopsies of 21 COVID-19 patients, lymphocytic myocarditis was present in 3 (14%) and increased interstitial macrophage infiltration was present in 18 (86%) of the cases [117]. There is still not enough evidence supporting the direct destruction of cardiomyocytes through virus-mediated lysis. Other mechanisms involving direct entry of the virus into endothelial cells in the heart or hyperactivation of the immune system have been proposed. Some authors suggest EMB should not be routinely performed due to scarce evidence of histological evidence typical for myocarditis, and actual virus RNA in cardiac tissue together with unclear therapeutic implications [118].

### 3.5. Takotsubo Cardiomyopathy

As already mentioned, myocardial injury in COVID-19 can be non-ischemic, i.e., stress-induced. Takotsubo cardiomyopathy or takotsubo syndrome (TTS), reversible myocardial dysfunction that can sometimes lead to acute heart failure, is usually caused by a stressful event, physical or psychological. In COVID-19 excessive catecholamine surge due to anxiety and immune response can result in typical and atypical myocardial stunning [43]. The prevalence of TTS in COVID-19 is not known yet, but there were several cases described [119,120,121,122,123]. There are some indications that the prevalence of TTS during the COVID-19 pandemic is rising, irrelevant of SARS-CoV-2 patient status. A retrospective cohort study, which included 1914 patients presenting with ACS in the Cleveland Clinic health system, showed a significant increase in the incidence of TTS during the COVID-19 period compared with the pre-COVID-19 period (7.8% vs. 1.5–1.8%). Because all the patients with TTS in the COVID-19 period were SARS-CoV-2 negative, the authors suggested that psychological, social, and economic distress accompanying the pandemic could be the factors associated with the increase in TTS cases [124].

### 3.6. Heart Failure in COVID-19

Heart failure (HF) in COVID-19 can be the consequence of myocardial injury, ACS, TTS, acute myocarditis, and arrhythmias. Moreover, HF can be caused by deterioration of preexisting myocardial dysfunction or unmasking of subclinical HF in individuals with underlying risk factors. It is a relatively common complication noted in 49% of patients who had severe/critical COVID-19 [125]. Similarly, in a retrospective, multicentre cohort study including 191 COVID-19 hospitalized patients, HF was developed in 23% of patients, 52% of non-survivors and 12% of survivors [126]. It seems that in COVID-19 heart failure with preserved ejection fraction (HFpEF) is more common than heart failure with reduced ejection fraction (HFrEF), both in the acute phase and chronic recovery phase [127]. Echocardiography study showed that in COVID-19 left ventricular systolic function is preserved in the majority of the patients (90%). Right ventricular abnormalities (dilatation and dysfunction) were found in 39%, and left ventricular diastolic dysfunction in 16% of patients [128]. NT-proBNP is a biomarker of hemodynamic myocardial stress and HF. It is frequently elevated among patients with severe/critical COVID-19, and according to some authors related to poor prognosis and increased mortality [88]. Current European Society of Cardiology Guidance for the Diagnosis and Management of CV Disease during the COVID-19 pandemic suggests a careful interpretation of NT-proBNP levels. They should be seen as the combination of the presence or extent of preexisting cardiac disease and/or the acute hemodynamic stress related to COVID-19. It should also be taken into account that NT-proBNP levels can be associated with the right ventricular hemodynamic stress, especially in the setting of VTE [18].

### 3.7. Venous Thromboembolism (VTE)

Hypercoagulability, supported by inflammation, activated coagulation cascade and immobilization, is a common manifestation of COVID-19 and increases the risk of thromboembolic events, such as deep vein thrombosis and pulmonary embolism, especially in critically ill patients [40]. The alveolar damage and the pulmonary microvascular thrombosis contribute to acute lung injury and respiratory dysfunction in COVID-19. According to some reports, autopsies performed in COVID-19 patients found thrombus formation in small and mid-sized pulmonary arteries despite the absence of clinical indicators of thromboembolism, while another series of autopsy findings showed an approximately 9 times higher incidence of pulmonary microvascular thrombosis compared to influenza cases [103,111,129]. As reported in one study, 31% of patients treated in ICU developed thrombotic complications despite prophylactic anticoagulation, and these thrombotic events were associated with 5.4 times higher risk of mortality [130]. Another study reported the prevalence of thrombosis in asymptomatic lower limbs by ultrasonography of 25% in ICU treated patients [131]. Furthermore, Wang et al. reported a high risk of VTE in 40% of hospitalized patients with COVID-19 using the Padua model, while a study with 191 COVID-19 patients found that 50% of patients who died had evidence of coagulopathy and that D-dimer levels greater than 1000 μg/L were likely associated with fatal outcome [42,132]. The prevalence of venous thromboembolism and pulmonary embolism is likely underestimated since the access to diagnostics, primarily computed tomography, may be limited for critically ill patients. All this data support the fact that all critically ill patients should receive anticoagulation prophylaxis, preferably using low molecular weight (LMW) [40]. 

## 4. Cardiovascular Diagnostics of COVID-19

### 4.1. Biomarkers

Biomarkers that are being widely used in the diagnosis of cardiovascular diseases, such as cardiac troponin T/I (cTn), B-Type Natriuretic Peptide/N-Terminal B-Type Natriuretic Peptide (BNP/NT-proBNP) and D-dimers, have to be carefully interpreted according to patients’ symptoms, comorbidities and acute settings. These biomarkers are not specific for just one clinical entity, but rather give additional information to the clinician that has to be put in a jigsaw puzzle. There are still no biomarkers specific to COVID-19 patients with cardiovascular disease (Table 1).

Elevated cTn and D-dimer levels have been found more often in COVID-19 patients with severe illness who were admitted to the ICU and in non-survivors (cTn was elevated in 46% patients in the non-survivor group and in 1% in the survivor group; D-dimer was elevated in 81% patients in the non-survivor group vs. 24% in survivor group) [126].

NT-proBNP as a biomarker of hemodynamic myocardial stress is more often elevated in COVID-19 patients with a cardiac injury who have a higher risk of in-hospital mortality [87].

According to ESC Guidance for the Diagnosis and Management of CV Disease during the COVID-19 pandemic, the levels of cTn and NT-proBNP correlate with disease severity and mortality but should be interpreted as quantitative variables. Mild elevations (<2–3 times the times the upper limit of normal) should be seen as a consequence of preexisting cardiac disease, acute injury or stress related to COVID-19, and do not require any additional workup or treatment if there are no symptoms such as chest pain or ECG changes. Pre-test probability assessment and serial measurements of D-dimers may be useful in the selection of patients who would benefit from additional VTE-imaging or the use of higher than prophylactic doses of anticoagulation. Routine measurements of cTn and BNP/NT-proBNP are discouraged [18].

### 4.2. Electrocardiographic Changes in COVID-19

There are no specific ECG characteristics in COVID-19 patients that were found so far (Table 1). Because of that, the ECG diagnostic criteria for cardiac conditions should be the same in COVID-19 patients and the general population [18]. According to a study from Wuhan, China, the most common ECG features found in COVID-19 patients were ST-T abnormalities (40%) and arrhythmias (38%). They were also associated with increased odds of ICU admission [113]. 

### 4.3. Noninvasive Imaging

#### 4.3.1. Echocardiography

Transthoracic echocardiography (TTE) is the most used imaging modality for cardiac evaluation in COVID-19 as it is can be easily performed at the patient’s bedside, providing information about cardiac structure and function [133]. One survey showed that the most common indications for TTE among 1216 examined COVID-19 patients were suspected heart failure, cardiac biomarker elevation and right-sided heart failure (Table 1) [134]. An abnormal finding on TTE together with myocardial injury, rather than the myocardial injury alone, is associated with higher mortality [135]. Moreover, another study showed that the most common findings on TTE were right ventricular (RV) dilation and dysfunction in 39%, followed by left ventricular diastolic dysfunction in 16% and left ventricular systolic dysfunction in only 10% of COVID-19 patients examined within 24 h of admission [128]. A right ventricular impairment that is associated with poor outcomes may be due to extensive use of mechanical ventilation and positive end-expiratory pressures for ARDS patients, as well as hypercoagulability, which both cause damage to lung parenchyma and pulmonary microvasculature resulting in pulmonary hypertension [133,136]. According to a study that included 120 COVID-19 patients right ventricular longitudinal strain (RVLS) is a strong predictor of higher mortality in patients with COVID-19, as patients with impaired RV strain are more prone to ARDS development needing high flow oxygen therapy and mechanical ventilation [137]. In addition to that, right ventricular ejection fraction measured by three-dimensional echocardiography (3D-RVEF) showed an even higher predictive value over standard RV function parameters and RV free wall longitudinal strain (RVFWLS) that may help to identify patients at higher mortality risk [138]. It is also reported that almost all critically ill COVID-19 patients (98%) and most non-critical patients (78.3%) have a detectable reduction of global longitudinal strain (GLS) measured by two-dimensional speckle-tracking echocardiography, which shows that strain is a very sensitive indicator of myocardial injury [139]. The use of transesophageal echocardiography (TEE) has been discouraged since the outbreak of the COVID-19 pandemic because of aerosol production and increased risk of viral spreading, and alternative imaging modalities are recommended instead. Focused cardiac ultrasound study (FoCUS) performed at bedside is reasonable option for cardiovascular complications screening in COVID- 19 infection as it reduces exposure time and direct contact with the patient [18].

#### 4.3.2. Computed Tomography (CT)

The role of computed tomography (CT) in COVID-19 patients is important, especially chest CT for evaluation and progression of lung pathology but can also be useful in helping differentiate COVID-19 pneumonia and acute decompensated heart failure [133,140]. Cardiac CT should be used to rule out cardiac thrombus and in acute chest pain to rule out obstructive coronary disease and in cases of LVAD dysfunction [18]. CT-pulmonary angiography (CTPA) enables detection of pulmonary embolism, a common finding in COVID-19 infection with incidence ranging from 2.6 to 24% according to some studies [133]. Cardiac CT angiography (CTA) may be used instead of TEE for the exclusion of left atrial appendage thrombus, evaluation of endocarditis and paravalvular complications. Coronary CTA can be an alternative to invasive coronary angiography [133,141]. 

#### 4.3.3. Cardiac Magnetic Resonance (CMR)

Cardiac magnetic resonance (CMR) imaging is a very sensitive tool for identifying COVID-19-related myocardial injury, but also for detection of post-COVID-related long-term cardiovascular consequences (Table 1) [133,142]. CMR can help distinguish between ischemic and non-ischemic myocardial injury and is useful for the diagnosis of myocarditis showing myocardial edema with late gadolinium enhancement, inflammation, and fibrosis [133]. According to one study, changes in ventricular function were less common than changes in T1/T2 mapping. This may be explained by inflammation, which is the most common way of cardiac involvement. The most common diagnosis made by CMR in COVID-19 patients was myocarditis (40%). On the other hand, approximately 20% of patients with “cardiac” symptoms had a normal MR [114]. Another study showed that 78% of recently recovered COVID-19 patients had abnormalities on CMR, and 60% had evidence of myocardial inflammation, even in asymptomatic patients, indicating the need for investigation of possible long-term cardiovascular consequences of COVID-19 [142,143]. A similar study reported evidence of myocardial inflammation or prior myocardial injury on CMR after either asymptomatic or mildly symptomatic COVID-19 in competitive athletes [144]. Therefore, CMR can be potentially used for making exercise recommendations post-COVID-19 [133].

#### 4.3.4. Nuclear Imaging

Nuclear imaging requires longer acquisition times and exposure of both patients and health care providers. The use of positron-emission tomography (PET-CT) can be an alternative to TEE for patients with suspected endocarditis of prosthetic valves or intracardiac devices (Table 1) [18,133]. 

### 4.4. Exercise Testing

Exercise testing is discouraged in the COVID-19 pandemic because of its serious limitations such as wearing a mask during the performance (Table 1). Still, it remains the method of choice for the indication to heart transplantation in patients with heart failure and for the diagnosis of heart failure with preserved ejection fraction (HFpEF) [18,145].

## 5. COVID-19 Treatment Strategies

### 5.1. Current Best Practice

Currently, there are different medications administered or under evaluation for use in treatment of SARS-CoV-2 infection, including antiviral, anti-inflammatory agents and immunosuppressants, increasing the risk of possible adverse effects or drug-drug interaction, especially in patients with chronic treatment for preexisting cardiovascular comorbidities or cardiovascular manifestation of COVID-19 requiring adequate guideline-directed pharmacotherapy (acute coronary syndrome, heart failure, myocarditis, arrhythmias and coagulopathy) [146,147]. According to the National Institutes of Health COVID-19 Treatment Guidelines (last updated on 21 April 2021), chronic therapy in COVID-19 patients such as ACEIs, ARBs, statins, systemic or inhaled corticosteroids, nonsteroidal anti-inflammatory drugs and acid-suppressive therapy should not be discontinued during acute management of COVID-19 [7].

As already discussed, SARS-CoV-2 infection may lead to excessive inflammatory response, platelet activation and endothelial disfunction, which then predisposes those patients to thrombotic events, both in venous and arterial circulation.

According to the COVID-19 Treatment Guidelines Panel, chronic anticoagulation or antiplatelet therapies should be continued in COVID-19 patients, unless there are contraindications, such as active bleeding or may be changed to shorter acting agents whose effect can be rapidly reversed if needed. Furthermore, COVID-19 patients not requiring hospitalization should not be given anticoagulation or antiplatelet therapy for prevention of venous thromboembolism. Prophylactic rather than therapeutic dose anticoagulation is appropriate for hospitalized COVID-19 patients, preferably low molecular weight heparin since clinical trials have not yet demonstrated a clear benefit of a higher dosing regimen. In case of severe renal impairment or renal replacement therapy, unfractioned heparin may be used, while fondaparinux is preferred in patients with heparin-induced thrombocytopenia. In COVID-19 patients with strongly suspected thromboembolism, thrombolytic therapy may be an option, preferably tenecteplase due to low bleeding risk and single-dose administration strategy [7,148].

When considering antiplatelet therapy with P2Y12 inhibitors and possible drug interactions with protease inhibitors, prasugrel is recommended in patients receiving lopinavir/ritonavir treatment, while clopidogrel is preferred in patients with coagulopathy due to lowest bleeding risk. Glycoprotein IIb/IIIa inhibitors should generally be avoided in COVID-19 patients [146].

As already mentioned in this review, since there is no evidence of possible harmful effect of ACEi and ARBs in COVID-19 patients, ESC Guidance for the Diagnosis and Management of CV Disease during the COVID-19 pandemic recommends continuation of antihypertensive treatment if indicated [18]. ACEi are not involved in significant cytochrome P450 (CYP450)-mediated metabolism so their interaction with antiviral agents is unlikely, while in coadministration with lopinavir/ritonavir decreased effect of losartan and irbesartan can be expected [149].

Loop diuretics can be safely administered in patients with COVID-19, while caution is required with mineralocorticoid receptor antagonists in patients receiving antiviral treatment that may increase the concentration of eplerenone, which then favors the use of spironolactone in these patients. For the same reason, coadministration of indapamide, ivabradine and digoxin with lopinavir/ritonavir should be avoided or requires closer surveillance [146,150].

When it comes to beta-blockers, beta-1 selective beta-blockers, mainly metoprolol and bisoprolol, are preferred in COVID-19 patients. With concomitant use of protease inhibitors, via involvement of the CYP450, there is a higher risk of bradyarrhythmias and hypotension which may require beta blocker dose down titration or drug cessation [146,149]. 

Statins are useful in primary and secondary cardiovascular prevention not only for their lipid lowering effect, but also anti-inflammatory effects which may explain their potential benefit in patients with acute viral respiratory infections. Since there are still no evidence of potential harm in COVID-19 patients, statin therapy should be continued with periodical liver function monitoring and advisable dose reduction in patients receiving lopinavir/ritonavir treatment [150,151].

Since cardiovascular complications are not rare in COVID-19 patients, pharmacotherapy needs to be tailored based on patients’ comorbidities (Table 2). Moreover, possible drug interactions between cardiovascular treatment and antiviral agents should be considered [146]. Arrhythmological considerations should be considered if antiviral medications such as chloroquine, hydroxychloroquine, azithromycin, lopinavir/ritonavir are used for acute COVID-19 management because of their impact on atrioventricular conduction, QTc prolongation and potential TdP risk. Identifying risk factors and correcting the modifiable ones, such as hypocalcemia, hypokalaemia and hypomagnesemia, together with reevaluation of concomitant therapy with drugs that have a bradycardic effect (e.g., beta-blockers, digoxin, ivabradine) are crucial [18]. Treatment strategies during the COVID-19 outbreak are based on early patient stratification according to the immediate health risk. According to the ESC Guidance, patients should be divided into one of the subgroups (Emergency, Urgent, Low priority, Elective) regarding their clinical condition [18]. Invasive cardiac procedures should not be postponed for the patients in the Emergency group who suffer from myocardial infarction (STEMI or very high/high risk NSTE-ACS), cardiogenic shock, symptomatic atrioventricular block, symptomatic sinus node dysfunction with asystolic pauses or cardiac tamponade. Furthermore, surgery in aortic dissection, cardiac trauma or acute failing of a native or prosthetic valve causing shock should not be postponed. Other invasive procedures can be done within days (in the Urgent group), within 3 months (Lower priority group) and after more than 3 months (Elective group). 

### 5.2. Emerging Treatment Options (MSCs)

Mesenchymal stem cells can be derived from various tissues, all of which are able to produce a strong paracrine effect in vivo that has an anti-apoptotic, anti-scarring, antibiotic, immunomodulatory and regenerative effect [152,153]. Previously, systemic MSC application was used to treat various degenerative and immune diseases across medical specialties, including cardiology [154]. Meta-analysis of MSC treatment for heart failure demonstrated a 36% mortality reduction, 34% readmission incidence, left ventricle ejection fraction (LVEF) increase of more than 5.25% in a total of 612 patients that were included [155]. In another meta-analysis of patients with ischemic heart disease, LVEF was increased by 3.84%, which was maintained 24 months after treatment, scar formation was reduced 6 months after treatment, while there was no statistically significant difference between mortality rates and hospital readmission incidence in MSC group compared to the placebo group. A total of 950 patients were included in this meta-analysis [156]. Knowing that the overaggressive immune response is one of the key features in patients who have severe COVID-19, MSC therapy was used for its immunomodulatory effects. A number of clinical trials are underway to evaluate the effect of MSC therapy for COVID-19, however, the available evidence at the time of writing this review indicates an overall positive effect of MSC treatment for patients with severe COVID-19 patients, including those who developed ARDS [157,158,159,160,161,162,163,164]. A combined positive effect for COVID-19 patients who have cardiovascular comorbidities is yet to be evaluated.

## 6. Long-Term Cardiovascular Effects of COVID-19

**Figure 2:**
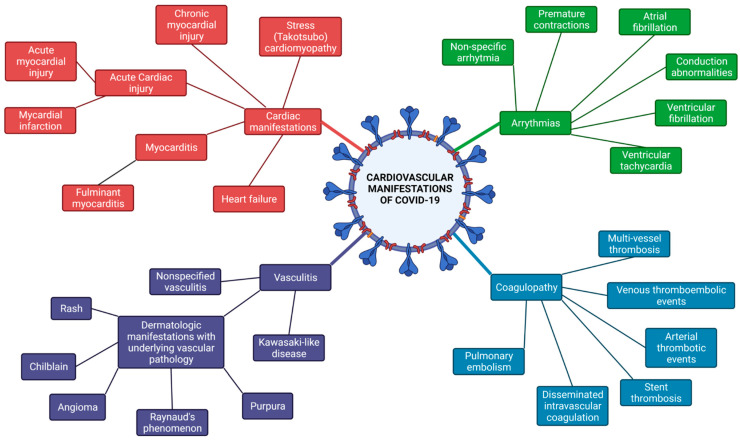
Schematic representation of COVID-19 related cardiovascular manifestations. Created in Biorender.com (accessed on 6 November 2021).

The persistence of symptoms in patients recovered from COVID-19 is common. A single-center study from Italy among 143 hospitalized patients recovered from COVID-19 found that 87.4% of them reported the persistence of at least one symptom, most commonly dyspnea and fatigue, even 60 days after acute illness onset, and 44.1% of patients reported worsened quality of life [165]. Almost one-third of patients hospitalized with COVID-19 have elevated troponin levels as a marker of myocardial injury, which is associated with poorer outcomes [166]. Since cardiovascular implications in SARS-CoV-2 infection are numerous given the multiple possible pathophysiological mechanisms underlying myocardial injury, both ischemic and non-ischemic, in the acute phase of the disease, potentially long-term cardiovascular effects are anticipated. These could include entities such as heart failure, Takotsubo syndrome, arrhythmias, sudden cardiac death, hypertension, reno-cardiac syndrome, accelerated atherosclerosis and both chronic venous and arterial thromboembolic disease [167]. Myocardial involvement in COVID-19 can trigger inflammation leading to possible myocardial fibrosis, which to a greater extent may be responsible for electrophysiological disturbances predisposing patients to atrial fibrillation and ventricular arrhythmias (Figure 2). Clinical manifestations of COVID-19 are diverse and hard to predict depending on several factors, including the mechanism of myocardial injury, severity of acute illness, immune system response and host reaction. Furthermore, it is not excluded that the treatment given in the initial phase of the disease may affect future cardiovascular complications [166]. Identifying COVID-19 survivors with subclinical or manifest myocardial disease and giving optimal treatment is important to lower the burden of long-term morbidity and mortality and emphasizes the importance of a proactive and careful approach in follow-up after hospital discharge. Further research is needed to better evaluate the so-called post-COVID-19 cardiac syndrome and to optimize patient screening and management [166,167].

## 7. Conclusions

Our knowledge of the cardiovascular impact of COVID-19 is evolving rapidly; onetheless, it is clear now that SARS-CoV-2 infection has major implications for the cardiovascular system. Regardless of the precise mechanism of cardiac damage caused by COVID-19, troponin values bring invaluable prognostic value in predicting mortality and clinical severity of the disease. Acute non-ischemic myocardial injury due to primary cardiac illness can occur in COVID-19 patients presenting with myocarditis, stress cardiomyopathy and acute heart failure. Surprisingly, recent studies suggest that the myocarditis incidence is significantly lower (less than 1%) than originally thought. However, cardiovascular consequences of COVID-19 extend the acute phase of infection. The increasing body of evidence suggests that chronic endothelial dysfunction is the crucial pathophysiological mechanism leading to a severe course of COVID-19 and worse patient outcomes. Hypercoagulability, supported by inflammation, activated coagulation cascade and immobilization, is a common manifestation of COVID-19 and increases the risk of thromboembolic events, such as deep vein thrombosis and pulmonary embolism, especially in critically ill patients. However, there are still no biomarkers specific to COVID-19 patients with cardiovascular disease. Biomarkers that are being widely used in the diagnosis of cardiovascular diseases, such as cardiac troponin T/I (cTn), B-Type Natriuretic Peptide/N-Terminal B-Type Natriuretic Peptide (BNP/NT-proBNP) and D-dimers, have to be carefully interpreted according to patients’ symptoms, comorbidities and acute settings. Noninvasive imaging to diagnose COVID-19 includes echocardiography, CT, CMR, nuclear imaging, and exercise testing. Treatment strategies during the COVID-19 outbreak are based on early patient stratification according to the immediate health risk. The European Society of Cardiology ESC Guidance for the Diagnosis and Management of CV Disease during the COVID-19 pandemic provided a guidance document covering all aspects of cardiovascular care during the COVID-19 pandemic. Nevertheless, the recent development of new technologies, including treatment with mesenchymal stem cells, is shedding light on novel therapeutic strategies.

## Figures and Tables

**Table 1:** Diagnostic approach for the cardiovascular complications of COVID-19 and their characteristic findings.

Diagnostic Modality	Indication/Characteristic Findings
Biomarkers	There are still no biomarkers specific to COVID-19 patients with cardiovascular disease. cTn, BNP/NT-proBNP and D-dimers are widely used but are not specific to just one clinical entity. The levels of cTn and NT-proBNP correlate with disease severity and mortality. Serial measurements of D-dimers may be useful in the selection of patients who may need additional VTE-imaging or the use of therapeutic doses of anticoagulation.
Electrocardiogram (ECG)	There are no specific ECG characteristics in COVID-19 patients. Atrial fibrillation is the most common tachyarrhytmia. Severe sinus bradycardia and complete heart block are the most common bradyarrhythmias. Monitoring-higher risk for prolonged QT and TdP when on COVID-19 drug therapy.
Exercise test	Discouraged in the COVID-19 pandemic (wearing a mask during exercise).
Echocardiography	The most common indications are suspected heart failure and elevated cardiac biomarkers. The most common findings on TTE are right ventricular dilatation and dysfunction. The use of TEE has been discouraged because of aerosol production. FoCUS is reasonable option for cardiovascular complications screening.
Computed tomography (CT)	Used for evaluation and progression of lung pathology. Useful for differentiation of COVID-19 pneumonia and acute decompensated heart failure. May be used instead of TEE for the exclusion of LAA thrombus, evaluation of endocarditis and paravalvular complications. Possible alternative for invasive coronary angiography.
Cardiac magnetic resonance (CMR)	T2-weighted imaging, early gadolinium enhancement, and late gadolinium enhancement signal intensities are the preferred imaging modalities for identifying COVID-19-related myocarditis. Can help distinguish between ischemic and non-ischemic myocardial injury.
Nuclear imaging	Requires longer acquisition times and exposure. PET-CT can be an alternative to TEE for patients with suspected endocarditis.

BNP/NT-proBNP—B-Type Natriuretic Peptide/N-Terminal B-Type Natriuretic Peptide; cTn—cardiac troponin; FoCUS—focused cardiac ultrasound study; LAA—left atrial appendage; PET-CT—positron-emission tomography; TEE—transesophageal echocardiography; TTE—transthoracic echocardiography; TdP—torsades de pointes.

**Table 2:** Current therapeutic considerations and concerns with cardiovascular treatment in COVID-19.

Drug	Consideration		Concerns
Antiplatelets	Chronic antiplatelet therapy should be continued if no contraindications COVID-19 patients not requiring hospitalization should not be given antiplatelet therapy for prevention of VTE		Active bleeding Increased risk of bleeding with ticagrelor and decreased antiplatelet activity with clopidogrel in patients receiving lopinavir/ritonavir treatment
	P2Y12 inhibitors	Clopidogrel is preferred in patients receiving fibrinolytics Prasugrel is recommended with lopinavir/ritonavir treatment	
	Glycoprotein IIb/IIIa inhibitors	Should be avoided	Increased risk of bleeding
Anticoagulants	Chronic anticoagulation therapy should be continued if no contraindications COVID-19 patients not requiring hospitalization should not be given anticoagulation therapy for prevention of VTE Prophylactic dose of LMWH is recommended for hospitalized COVID-19 patients UFH may be considered in case of severe renal impairment or RRT Fondaparinux is preferred in patients with HIT		Increased risk of bleeding DOACs not recommended with lopinavir/ritonavir treatment
ACEi/ARB	Chronic therapy should be continued if indicated		Decreased effect of losartan and irbesartan can be expected in patients receiving lopinavir/ritonavir treatment
Diuretics	Loop diuretics can be safely administered		Concentration of indapamide increases with lopinavir/ritonavir treatment
Mineralocorticoid receptor antagonists	Spironolactone is preferred		Increased risk of adverse effect with lopinavir/ritonavir treatment
Statins	Statin therapy should be continued with periodical liver function monitoring		Dose reduction should be considered in patients receiving lopinavir/ritonavir treatment
Beta-blockers	Beta-1 selective beta-blockers are preferred (metoprolol and bisoprolol)		Higher risk of bradyarrhythmias and hypotension with concomitant use of protease inhibitors
Antiarrhythmics	Monitor ECG and serum electrolyte levels before initiation		Increased risk of QT prolongation and TdP with chloroquine/hydroxychloroquine Increased antiarrhythmic effects with protease inhibitors

ACEi: angiotensin-converting enzyme inhibitor; ARB: angiotensin receptor blocker; DOACs: direct oral anticoagulants; HIT: heparin-induced thrombocytopenia.

## Data Availability

Not applicable.
